# A Coronary Cameral Fistula Draining Into Left Ventricle: A Rare Finding

**DOI:** 10.7759/cureus.25755

**Published:** 2022-06-08

**Authors:** Rajwinder Gill, Abhinav Sood

**Affiliations:** 1 Internal Medicine, Icahn School of Medicine at Mount Sinai Beth Israel, New York City, USA; 2 Cardiovascular Disease, Icahn School of Medicine at Mount Sinai Beth Israel, New York City, USA

**Keywords:** arterial luminal, coronary artery angiogram, angiogram, ccf, anomalous artery, coronary cameral fistula

## Abstract

Coronary-cameral fistulae (CCF) are abnormal connections between coronary arteries and cardiac chambers. CCF are mostly congenital but can be acquired as well. CCF draining into the left-sided chambers are very rare. We present a case of an incidentally detected CCF arising from the left anterior descending artery and draining into the left ventricle.

## Introduction

Coronary-cameral fistulae (CCF) are present in 1% of the population. CCF arising from the right coronary system are most common. Origin from the left coronary system is seen in 35% of cases and bilaterally in 5% of cases. Of the CCF, 99% drain into the right cardiac chambers [1]. Most of the cases are asymptomatic and detected as an incidental finding on coronary angiogram. Large CCF can lead to the symptoms. In infants, it can present with diaphoresis, tachypnea, and tachycardia during feeding. In adults, it can present with angina due to the coronary steal phenomenon, symptomatic heart failure, or a combination of both [2].

## Case presentation

A 77-year-old female with a history of hypertension, hyperlipidemia, hypothyroidism, Sjogren's syndrome, and anxiety was referred to outpatient cardiology for evaluation of new-onset intermittent exertional dizziness over the past five months without syncope. The patient had a family history of myocardial infarction in her mother at the age of 61. She had no family history of sudden cardiac death. As per home medications, she was on rosuvastatin 5 mg, levothyroxine 50 mcg, lisinopril 5 mg, and alprazolam 0.25 mg.

Vital signs included heart rate of 84 beats/minute, blood pressure of 130/78, respiratory rate of 15 per minute, and oxygen saturation of 98% on room air. The cardiovascular exam was unremarkable for any murmur, pedal edema and lungs were clear to auscultation without any rales/wheezes/rhonchi. Laboratory work-up was unremarkable with normal troponin and brain natriuretic peptide (BNP). All the laboratory work is shown in Table 1. 

**Table 1:** Laboratory work-up pCO2: partial pressure of carbon dioxide

Test	Results	Reference Range
White Blood Count (WBC)	5.2	4.5-11.00 k/uL
Hemoglobin	14	13.6-16.3 G/DL
Platelet	288	150-450 k/uL
Sodium	139	135-145 meq/L
Potassium	4.3	3.5-5.2 mmol/L
Chloride	99	96-108 mmol/L
Phosphorus	2.6	2.4-4.7 mg/dL
Magnesium	2.2	1.5-2.5 mg/dL
Creatinine	0.71	0.5-1.1 MG/DL
Blood Urea Nitrogen	19	6-23 MG/DL
Brain Natriuretic Peptide (BNP)	31	0.0-100 pg/mL
Troponin	<0.010	<0.031 mg/dl
Aspartate Aminotransferase	22	1-35 U/L
Alanine Aminotransferase	26	1-45 U/L
Alkaline Phosphatase	66	38-126 U/L
pH	7.40	7.35-7.45
pCO_2_	42	35-45 mmHg
Bicarbonate	24	21-29 mEq/L
Lactic acid	0.9	0.50-2.00 mmol/L
Thyroid Stimulating Hormone (TSH)	1.645	0.4-4.2 uIU/mL

**Figure 1:**
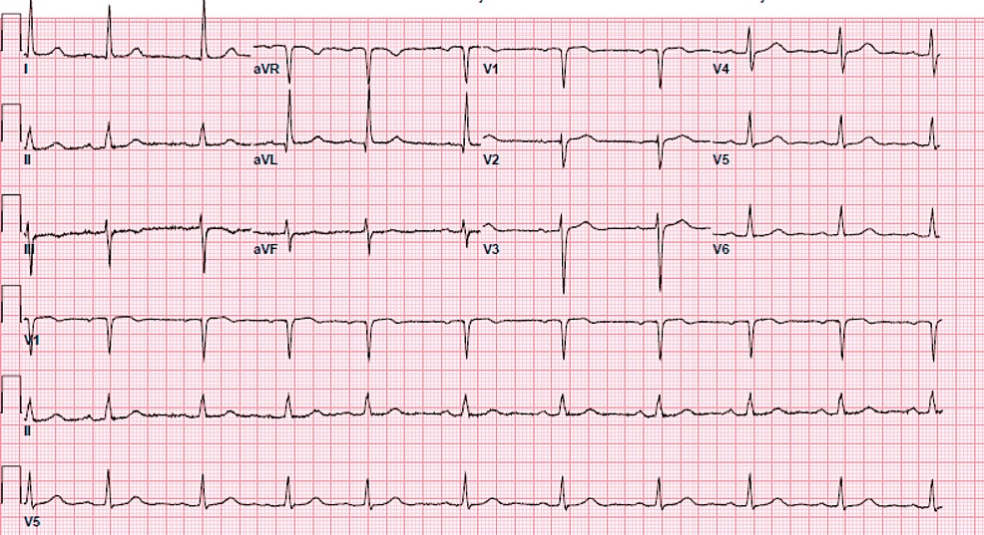
Electrocardiogram showing normal sinus rhythm

Electrocardiogram (ECG) showed normal sinus rhythm (Figure 1). Transthoracic echocardiogram (TTE) detected Grade 1 left ventricular diastolic dysfunction without any left atrial enlargement, mild mitral regurgitation, tricuspid regurgitation, and aortic regurgitation. Exercise stress echocardiogram showed very poor functional capacity and anteroseptal hypokinesis suggesting ischemic heart disease for which coronary angiography was pursued. She was sent back on cardiac monitoring because of dizziness.

**Figure 2:**
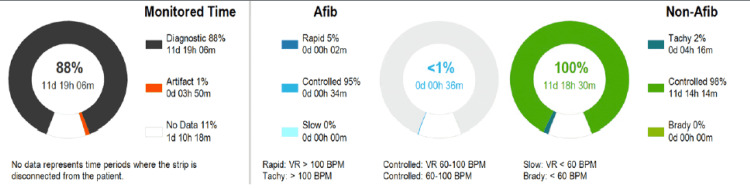
Cardiac monitoring done for 14 days showing atrial fibrillation AFib: atrial fibrillation; BPM: beats per minute; d: days; h: hours; m: minutes

Fourteen days of cardiac monitoring showed multiple runs of atrial fibrillation with a burden of less than 1%. The patient was started on apixaban 5 mg and metoprolol succinate 25 mg. Cardiac monitoring report is shown in Figure 2. 

Coronary angiography was done via right radial approach and showed mild obstructive coronary artery disease with no focal stenosis and anomalous connection between a left anterior descending artery and left ventricle, which is shown in Video 1. Left ventricular end-diastolic pressure (LVEDP) at the time of angiography was 12 mmHg. Her symptoms improved after starting on beta-blocker. 

**Video 1:** Coronary angiogram after injection to left main artery showing the contrast in left ventricular cavity

## Discussion

Epidemiology and classification

CCF can be classified based on etiology or morphology. With regards to etiology, CCF are either congenital or acquired. While the majority of cases are congenital, acquired causes are cardiac surgery, invasive procedures like coronary angiogram, pacemaker implantation, and trauma [3].

In terms of morphology, there are three types of CCF: arterial luminal(most common), arterio-sinusoidal, and arterio-capillary fistula. Arterial-luminal is a direct connection between a coronary artery and the cardiac chamber. In the second form, the arterio-sinusoidal type, the communication is through the myocardial sinusoidal network. In the arterio-capillary fistula, the arterial vessels drain into the capillaries and then through the small cardiac veins, known as Thebesian veins, into the heart chamber [4].

Pathophysiology

CCF is most often an incidental finding and is usually asymptomatic in the majority of cases. CCF draining into the left ventricle may produce a diastolic murmur and can increase the diastolic filling, mimicking aortic valve insufficiency [5]. Arterio-luminal type and arterio-sinusoidal type can lead to angina pectoris by bypassing the vessels for oxygenated blood delivery to the myocardium. Angina pectoris would be very unlikely in arterio-capillary fistula. Large communication can lead to heart failure in the later stages of life due to the constant volume overload to the ventricle. Rupture of the aneurysm and infective endocarditis are also reported in a few cases [4]. 

Diagnosis and management

Coronary angiography and/or cardiac computed tomography are the two modalities used to diagnose CCF [6]. The management of coronary artery fistulae is controversial and depends on presentation. The natural history of CCF is rather unclear in the current literature. As per the current literature, CCF in patients with heart failure symptoms with large communications should be closed [6]. CCF in symptomatic patients with smaller fistulas should be closed early as they tend to get large with age. Elective closure of CCF is also recommended in asymptomatic patients with a continuous murmur or systolic murmur with an early diastolic component [7]. Arterio-luminal fistulas can be closed with surgery but arterio-sinusoidal can’t be closed with surgery. Literature shows symptomatic improvement with beta-blockers in a few cases [2].

CCF closure can be done with cardiac catheterization or surgically. Although there is less morbidity and mortality associated with surgical technique, cardiac catheterization is a preferred technique. The different percutaneous catheter techniques include Gianturco coils, interlocking detachable coils, detachable balloons, polyvinyl alcohol foam, double umbrellas, the Amplatzer™ Duct Occluder (Abbott Laboratories, Chicago, Illinois, United States), and Amplatzer Vascular Plugs (Abbott Laboratories). These closure devices can sometimes migrate into the extracoronary vessels or even in coronary arteries and can lead to myocardial infarction occasionally [7].

## Conclusions

In this case, there was a small CCF. Exertional dizziness was most likely due to atrial fibrillation. Although stress echocardiogram findings were suggestive of ischemic heart disease, coronary angiography revealed diffuse coronary artery disease without any focal stenosis.
